# Safety and efficacy of intralesional steroid injection for aggressive fibromatosis

**DOI:** 10.1186/s12957-017-1262-9

**Published:** 2017-11-02

**Authors:** Dumnoensun Pruksakorn, Sratwadee Lorsomradee, Areerak Phanphaisarn, Pimpisa Teeyakasem, Jeerawan Klangjorhor, Parunya Chaiyawat, Natapong Kosachunhanun, Jongkolnee Settakorn, Olarn Arpornchayanon

**Affiliations:** 1 Orthopedic Laboratory and Research Network (OLARN), Department of Orthopedics Faculty of Medicine, Chiang Mai University, Chiang Mai, Thailand; 2 Excellence Center in Osteology Research and Training Center (ORTC), Chiang Mai University, Chiang Mai, Thailand; 3 Department of Anesthesiology, Faculty of Medicine, Chiang Mai University, Chiang Mai, Thailand; 4 Endocrinology Unit, Department of Internal Medicine, Faculty of Medicine, Chiang Mai University, Chiang Mai, Thailand; 5 Department of Pathology, Faculty of Medicine, Chiang Mai University, Chiang Mai, Thailand; 6 Bangkok Hospital Chiang Mai, Chiang Mai, Thailand

**Keywords:** Aggressive fibromatosis, Desmoid, Injections, Intralesion, Steroids

## Abstract

**Background:**

Treatment of recurrent aggressive fibromatosis (AF) following surgical resection is a clinical challenge. Non-steroidal anti-inflammatory drugs (NSAIDs) have been reported to be an effective option for controlling the disease. However, long-term NSAID use can result in unfavorable complications. This study was a trial of the use of intralesional steroid injection (ILSI) including investigation of safety margins and clinical outcomes of high-dose steroids for local use treatment of AF.

**Methods:**

A prospective cohort study was conducted to evaluate the safety and efficacy of particulate corticosteroids for AF. Intralesional steroid injections of Kanolone® guided by ultrasound were given monthly for three consecutive months with 1 mg/kg/episode (a total of 3 mg/kg). Patients were followed up monthly for 3 months at the time of each monthly injection and then for an additional 3 months after the last injection. Complications from the procedure and clinical outcomes were monitored.

**Results:**

Eight recurrent AF patients completed the full 6-month evaluation process. No procedure-related complications were reported either during the injection period or the follow-up period. None of the patients developed Cushingoid features. The highest number of complication events, all of which were mild or detectable only by laboratory analysis, occurred during the month following the second injection. Triamcinolone levels were significantly increased 24 h after injection, and four of the eight cases developed hypothalamic-pituitary-axis suppression. Tumors were stabilized in 83.3% of the cases during the study period, and pain and functional ability scores improved significantly.

**Conclusions:**

Intralesional steroid injection appears to be a safe and effective alternative treatment for recurrent AF.

**Trial registration:**

TCTR20150409001; Registered date: 9 April 2015; The safety and result of intratumoral steroid injection for aggressive fibromatosis.

**Electronic supplementary material:**

The online version of this article (10.1186/s12957-017-1262-9) contains supplementary material, which is available to authorized users.

## Background

Aggressive fibromatosis (AF) is a fibroblast proliferation that is intermediate in their biological behavior between superficial fibromatosis and fibrosarcoma [1]. Clinical behavior of AF is heterogenous and characteristically unpredictable. A multimodality approach, including surgery, radiation, chemotherapy, medication, and hormonal therapy, is a treatment option for local control [2]. Surgery is generally accepted as the treatment of choice for newly diagnosed AF, while non-surgical procedures have been frequently used in cases of recurrent and unresectable tumors [3]. The idea of using non-steroidal anti-inflammatory drugs (NSAIDs) for fibromatosis resulted from the surprising observation of total regression of a recurrent AF when taking indomethacin [4]. Following that observation, other types of NSAIDs have been studied for disease response. However, long-term use of NSAIDs and the accompanying complications remain a primary concern. Triamcinolone acetonide, a particulate corticosteroid preparation, is commonly used in treating the musculoskeletal system. Local steroid injection is widely used to reduce local inflammation of various tissues and has also been accepted for controlling the progression of hypertrophic scar [5]. The corticosteroid particles accumulate in the local tissue and are slowly released, achieving long-term control of inflammation in the local area.

Although surgery is the mainstay treatment for newly diagnosed fibromatosis, resection of recurrent tumor becomes a clinical challenge in optimizing between surgical margin and morbidity. On the other hand, controlling pain, stabilizing the tumor, and maintaining function are the major concerns for non-metastasis tumor. In this study, intralesional steroid injection (ILSI) was studied in patients who had a recurrence of AF. The primary objective of the study was to monitor complications and clinical outcomes following ILSI treatment.

## Methods

### Patient enrollment

The protocol for use with the prospective cohort to evaluate the safety and efficacy of corticosteroid by ILSI in recurrent AF was approved by the Ethics Committee of the Faculty of Medicine, Chiang Mai University (ORT-12-1184-FB(ID:1184)), and was registered with the Thai Clinical Trials Registry (TCTR20150409001). Ten patients were initially enrolled. Inclusion criteria were diagnosis with AF by the Musculoskeletal Oncology Board of Chiang Mai University, at least one episode of recurrence of the disease after wide resection, and either a progressively enlarging or a symptomatic tumor. Exclusion criteria were bleeding tendency, dependence on systemic steroid use for other conditions and contra-indications for steroid use including poorly controlled diabetes, an active infection, pregnancy, and lactation. All participants gave their written consent to participate in the research.

### Treatment and follow-up protocols

**Fig. 1:**
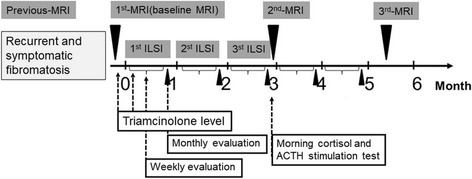
Operational diagram of ILSI for recurrent fibromatosis

Patients were admitted to the Orthopedic Department, Faculty of Medicine, Chiang Mai University Hospital for a period of 2 days. On the first day, interviews and laboratory examinations were conducted followed by the 1st-ILSI. Patients remained in the hospital for a second day after the ILSI to allow monitoring of possible acute complications. During the procedure, a spinal needle was inserted into the tumor using an ultrasound guide. Five mg/mL (1:1 dilution with normal saline) of 10 mg/mL Kanalone®, triamcinolone acetate was injected. The amount of triamcinolone acetate was 1 mg/kg/episode but not over 80 mg in a single episode. Treatment was repeated monthly (every 4 weeks) for three consecutive months, a total dose of 3 mg/kg in 3 months, with no repeat dose within 1 year of the final injection [6]. After discharge from the hospital, a nurse practitioner from the Clinical Trial Unit interviewed patients by phone weekly regarding procedure-related complications and symptoms of Cushing’s syndrome (weekly evaluation: Fig. 1). Nine types of complications were reported during the subjective phone interviews: irregular menstruation, swelling of extremities, muscle weakness or pain, insomnia, mood instability, gastrointestinal disturbance, hirsutism, headache, and flu-like feelings. Four examination parameters (blood pressure, pulse rate, respiratory rate, and temperature) and eight laboratory parameters (fasting blood sugar, BUN, Cr, Na, P, Cl, CO_2_, and WBC count) were evaluated just prior to each subsequent injection (monthly evaluation: Fig. 1). Blood pressure and blood sugar levels were evaluated following standard guideline [7, 8]; abnormalities in laboratory parameters, those outside the normal range, were noted. Triamcinolone levels were measured just prior to and 24 h after the first injection. Enzyme-linked immunosorbent assay was conducted using a triamcinolone ELISA kit (Neogen, US). Morning cortisol level was measured 1 month after the final ILSI. Patients with an inconclusive morning cortisol level (5–8 μg/dL) were given the ACTH stimulation test using a 250 μg cosyntropin injection [9]. Criteria for discontinuation of ILSI were patient declined to undergo a procedure and patient presented with systemic and local complications which were determined to be major complications. Treatment intervention and follow-up protocols are summarized in Fig. 1.

### Pain and functional ability evaluation

Pain and functional ability were measured before the first injection and then every 4 weeks just prior to the subsequent injection (monthly evaluation, Fig. 1). Patients were asked, “What was your overall pain/functional level during the month following the last injection?” Pain scores were evaluated using the Numeric Rating Scale for Pain (NRS pain) [10]. Functional ability was assessed by comparing responses after treatment with responses on previous questionnaires regarding functional level either before tumor growth in the same extremity or the functional level in the opposite extremity. A 4-point rating scale of functional level was used, ranging from normal (0) to the worst experience (3).

### Tumor progression evaluation

Tumor response monitoring protocol was designed based on the RECIST guideline [11]. The tumor was observed with magnetic resonance imaging (MRI) 1.5 Tesla, GE Healthcare, using the technical protocols for fibromatosis. The targeted lesion was evaluated by musculoskeletal radiologists. The size of the tumors was measured at the widest dimension in sagittal, coronal, and cross-sectional view. MRI was performed 3 months before the initial injection and was repeated just prior to that injection (baseline MRI). MRI was performed again 1 month after the third injection (the third month after baseline MRI), and again 3 months after the third injection (the sixth month after baseline MRI), Fig. 1. Tumor response was defined by summation of the three axis, and tumor evaluation was compared with baseline MRI. Complete response(CR) was defined as disappearance of the targeted lesion, partial response(PR) was defined as at least a 30% decrease in the sum of diameters, progressive disease(PD) was defined when at least a 20% increase in the sum of the diameters, and stable disease(SD) was defined as neither sufficient shrinkage to qualify as PR nor sufficient increase to qualify as PD.

### Statistical analysis

Pain scores and functional ability scores of both pre- and post-injections were compared using the non-parametric Wilcoxon signed-rank test. The relation between the dose of corticosteroid used and serum triamcinolone level was evaluated using the Pearson’s correlation coefficient. All statistical analyses were performed using STATA version 12.1. Statistical significance was considered to be *p <* 0.05.

## Results

### Demographic data

Ten recurrent AF patients participated in this study, of whom eight were able to comply to complete the full-evaluation processes. Cases number 1 and 10 were discontinued after the first and third injection, respectively, when they declined to participate in any invasive evaluations and declined to participate in the MRI tests. Patient demographic data and steroid doses received are shown in Table 1.

**Table 1:** Demographic data and initial evaluation

Case no.	Age	Gender	Dose (mg)‡	BMI (kg/m^2^)	Site of tumor	Treatment history
01†	44	Male	80	30.04	Lower back	Post-3rd surgery with S and SD
02	51	Female	47	18.83	Lower back	Post-1st surgery with S and PD
03	30	Female	52	21.64	Shoulder	Post-3rd surgery with S and PD
04	23	Male	68	23.53	Lower neck	Post-2nd surgery with S and PD
05	55	Female	58	25.78	Shoulder	Post-2nd surgery with S and PD
06	45	Female	53	20.70	Thigh	Post-3rd surgery with S and SD
07	18	Male	60	20.05	Shoulder	Post-1st surgery with S and PD
08	56	Female	65	24.77	Shoulder	Post-5th surgery with S and SD
09	40	Female	58	22.65	Forearm	Post-3rd surgery with S and PD
10†	20	Male	65	21.22	Thigh	Post-3rd surgery with AS and PD

*S* symptomatic disease including pain and/or discomfort, *AS* asymptomatic disease, *SD* stable disease, *PD* progressive disease

†Dropped out of study,

‡mg/episode

### Procedure-related and steroid-related complications

**Fig. 2:**
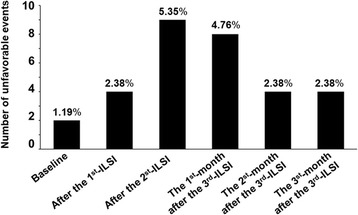
Percentage of unfavorable events out of 168 observations each month (21 observed parameters and 8 participants): nine items from subjective interviews, four items from examination parameters, and eight items from laboratory parameters

No procedure-related complications were identified either immediately after the injection or during the follow-up period. None of the participants developed Cushingoid features. All unfavorable events were mild or were detectable only by laboratory parameters. “Swelling of the extremity” was the only event reported in the subjective interviews. “High blood pressure” and “hypertension” were identified based on examination parameters, and “steroid-induced impaired fasting glucose” and “steroid-induced diabetes mellitus” were identified based on the laboratory parameters. The highest number of unfavorable events presented after the second ILSI, with the number decreasing in the following periods (Fig. 2). Three of the eight patients complained of swelling of extremities and fingers after the second ILSI and that symptom was gone by 1 month after the final injection, Additional file 1: Table S1. Three cases presented new findings of high blood pressure or hypertension. In one case, the high level of blood pressure continued until the end of the study. The two cases who had high blood pressure or hypertension before the procedure presented with obvious sign of hypertension in all courses of follow-up, Additional file 1: Table S2. Three individuals developed temporary steroid-induced impaired fasting glucose, and one developed steroid-induced diabetes mellitus after each injection, Additional file 1: Table S3. No patients presented with either abnormal electrolyte levels or abnormal WBC profiles during the treatment and subsequent monitoring period.

### Serum triamcinolone levels, morning cortisol levels, and ACTH stimulation test results

Triamcinolone levels were significantly higher 24 h after each injection, increasing to an average of 55.3 ± 15.2 ng/mL from baseline levels, Additional file 1: Figure S1. The amount of increase in triamcinolone level was significantly correlated with the dose of the injection (coefficient = 0.73, *p* = 0.04). One month after the final injection, four cases had normal cortisol levels. Three of those four cases were diagnosed based on comparison with normal levels of morning cortisol (cases no. 5, 7, 9), and one was diagnosed based on the ACTH-stimulation test which was conducted following an inconclusive morning cortisol result (case no. 4). Four cases presented with hypothalamic-pituitary-adrenal axis (HPA) suppression. One case was diagnosed with an abnormal level of morning cortisol (case no. 2). Three cases presented with HPA suppression after ACTH stimulation (cases no. 3, 6, 8), Additional file 1: Table S4. Mean steroid-using levels were higher in patients with HPA suppression (61.00 ± 4.76 mg/episode) than that in patients with normal HPA (54.25 ± 7.63 mg/episode), but the difference was not statistically significant because of the small sample size.

### Pain and functional ability scores and tumor progression

**Fig. 3:**
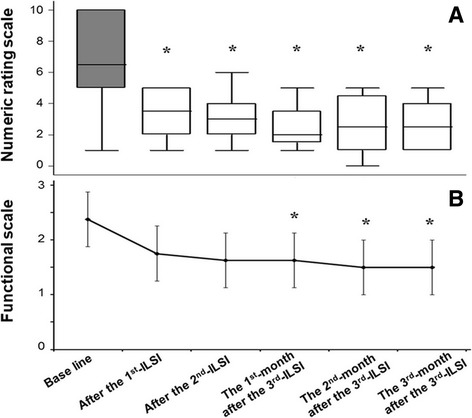
Numeric rating scale for pain of pre- and post-ILSI (**a**) and functional ability score of pre- and post-ILSI (**b**)

**Fig. 4:**
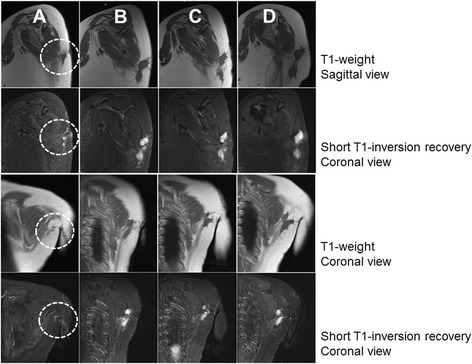
Representative MRI from case No. 3. Column **a**: sixth month after first surgical operation and three months before starting of the procedure. Column **b**: just prior to the first ILSI determined progression of tumor. Column **c**: tumor stabilization from baseline after the final protocol of ILSI. Column **d**: progression disease from baseline after the third month of ILSI

Pain scores were significantly improved in most of the cases (Fig. 3a). The reduction of pain was most distinct in patients who had initially had a high pain score. Overall functional ability was significantly improved in most of the cases. Patients reported feeling more comfortable in their daily activities (Fig. 3b). Tumors stabilized from progressive growth during treatment in five of the six cases (83.3%). Tumor stabilization continued beyond the final injection to the sixth month follow-up, in three of the six cases (50.0%). In two of six cases (25.0%), the tumor had progressively enlarged again by the sixth month after having stabilized during treatment. In one case (12.5%), there was no response to the treatment protocol and a tumor continued to grow during and after the treatment period, Table 2. Representative MRI sequential imaging is shown in Fig. 4.

**Table 2:** Tumor response by anatomical measurement

Case no.	Before injection†	After ILSI‡	The 3rd-month after ILSI§
02	Progressive disease	Stable disease	Stable disease
03	Progressive disease	Stable disease	Progressive disease
04	Progressive disease	Stable disease	Stable disease
05	Progressive disease	Stable disease	Progressive disease
06	Stable disease	Stable disease	Stabile disease
07	Progressive disease	Progressive disease	Progressive disease
08	Stable disease	Stable disease	Stable disease
09	Progressive disease	Stable disease	Stable disease

*ILSI* intralesional steroid injection

†Baseline MRI compared with the previous MRI

‡Compared with baseline MRI

§Compared with baseline MRI

## Discussion

Management of AF involves a wide range of modalities, while results of treatment remain unpredictable. Wide resection is the treatment of choice for primary fibromatosis; however, a recurrent rate is still high, with 1- and 5-year recurrence-free survival rate of 75–81.3% and 52.8–64%, respectively [12, 13]. With less invasive treatment, sulindac and high-dose selective estrogen receptor modulators (SERMs), tumors were stabilized or regressed in 85.1% of patients [14]. A wait-and-see policy was introduced for patients refusing surgery; of that group, 20% showed regression, 60% were stable, and 30% showed progression [15]. Surgical resection with adjuvant radiation for recurrent AF has been reported to be a better control method than that surgery alone, with local control between 78 and 32% at 6 year follow-up, respectively [16]. Use of radiation alone for recurrent AF showed local control rates of about 80 and 69% at 2 and 5 years, respectively [17]. Chemotherapy for unresectable AF showed a 52% partial response and significant improvement in pain and function [18]. A study of targeted therapy, using imatinib, reported a one-year progression-free survival of 66% and an objective response rate of 6%. In this study, grade 3 or 4 complication events occurred with a frequency of > 5% [19]. Intralesional steroid injection was able to stabilize around 83.3% of tumors and provided significant symptomatic relief in the majority of cases. The short-term outcomes suggest that a long-term effectiveness study would be appropriate. High-dose steroids result in a lower incidence and milder complications than other aggressive approaches. It is anticipated that a reduced dose and longer intervals between applications will be included in the next trial to minimize complications and maximize efficacy of this alternative procedure.

Aggressive fibromatosis presents with a wide variety of behaviors which have resulted in a lack of consensus regarding appropriate definitive management in either primary or recurrent cases. Because of the unpredictability of outcomes with each type of treatment, an individual approach has been recommended by some authors [20]. Spontaneous regression and stabilization from medications and from non-invasive treatment, described above, has been reported. Overall, it appears that generally, it would be best to initially select a treatment option which involves a less invasive procedure, which is generally available, which is suitable for long-term care, and which has a low level of complications. More invasive intervention (or medications) should be considered if the disease proves resistant to the less-invasive methods. Particularly in recurrent episodes, symptomatic control and maintenance of the capacity to perform normal daily activities have to be a major goal not just complete removal of the tumor.

Intralesional steroid injection is accepted as the standard procedure for keloid and hypertrophic scar management [21]. A similar histology finding would be interested to use the similar protocol to control AF. Achievement of good control of progressive palmar and plantar fibromatosis by using ILSI has been reported [22, 23]. A recent report by Holmes et al. describes a retrospective study of ten patients who received ILSI 0.5–2 mL of 10 mg/mL triamcinolone for symptomatic infantile digital fibromatosis with an average follow-up time of 5.8 years. The results showed that the recurrence rate of disease with ILSI was lower than with surgical treatment, and no complications due to the procedure were reported [24].

Recent studies of ILSI in keloid treatment have used a wide range of concentrations, from 10 to 240 mg for a single dose and from 40 to 1200 mg of total doses for repeated treatment. Occasional occurrence of Cushing’s syndrome resulting from having received steroids has been reported. A systematic review of treatment with triamcinolone found that use in children and at concentrations higher than 30 mg/month create a risk for Cushing’s syndrome [25]. Recommendations on appropriate dosages and frequency of administration have been limited because of the unpredictability of results and responsiveness of individuals to steroid use. The present study found that steroids are released into circulation in the following day, and the accumulated total dose causes subclinical HPA suppression in a half of series. The maximum dose recommendation, not over 54.25 mg per episode with three consecutive monthly episodes, has never resulted in HPA suppression. As long-term use of intralesional steroid injections can be anticipated for controlling the disease, smaller doses and longer intervals between administrations of steroids should be studied to help avoid unfavorable effects. Dosage used might be varied based on tumor size rather than total patient weight.

The procedure investigated in this study could be an option for non-invasive intervention to control disease progression. This method is potentially an appropriate alternative for long-term control of recurrent AF in patients who exhibit both good responsiveness and tolerance of steroids. In addition, longer term follow-up will be needed to evaluate responsiveness more conclusively.

## Conclusions

Intralesional triamcinolone stabilized tumors in about 83.3% of cases during the study period. All the patients tolerated the procedure well, with only a few minor complaints. Additional studies with a smaller dosage and involving more frequent injections and a longer follow-up monitoring of responsiveness are recommended.

## Additional file

Additional file 1: Table S1. Subjective interviews with patients regarding unfavorable side effects from steroid use. Swelling of extremities was the only positive presentation during or after the ILSI procedure. **Table S2.** Blood pressure change before and after procedure. **Table S3.** Fasting blood sugar before and after procedures. **Table S4.** Morning cortisol level and ACTH stimulation test. **Figure S1.** Serum triamcinolone level 24 h after intralesional steroid injection. (DOC 124 kb)

## Abbreviations

AF: Aggressive fibromatosis
HPA: Hypothalamic-pituitary-adrenal axis
ILSI: Intralesional steroid injection
MRI: Magnetic resonance imaging
NSAIDs: Non-steroidal anti-inflammatory drugs
SERMs: Suppression selective estrogen receptor modulators
