# Supplementary material for: Safety and efficacy of intralesional steroid injection for aggressive fibromatosis
Source: World J Surg Oncol. 2017 Nov 2;15:195. doi: 10.1186/s12957-017-1262-9 (PMC5667493; doi:10.1186/s12957-017-1262-9)
Supplement: Additional file 1: Table S1. — Subjective interviews with patients regarding unfavorable side effects from steroid use. Swelling of extremities was the only positive presentation during or after the ILSI procedure. Table S2. Blood pressure change before and after procedure. Table S3. Fasting blood sugar before and after procedures. Table S4. Morning cortisol level and ACTH stimulation test. Figure S1. Serum triamcinolone level 24 h after intralesional steroid injection. (DOC 124 kb) [file 12957_2017_1262_MOESM1_ESM.doc]

**Additional file**

**Table S1.** Subjective interviews with patients regarding unfavorable side effects from steroid use. Swelling of extremities was the only positive presentation during or after the ILSI procedure.

| Case No | Days 1-28  after the 1^st^-ILSI | Days 1-28  after the 2^nd^-ILSI | Days 1-28  after the 3^rd^-ILSI | 2^nd^ month  after the 3^rd^-ILSI | 3^rd^ month  after the 3^rd^-ILSI |
| --- | --- | --- | --- | --- | --- |
| 2 | None | None | None | None | None |
| 3 | None | None | None | None | None |
| 4 | None | None | None | None | None |
| 5 | None | **Swelling** | None | None | None |
| 6 | None | **Swelling** | None | None | None |
| 7 | None | None | None | None | None |
| 8 | None | **Swelling** | **Swelling** | None | None |
| 9 | None | None | None | None | None |

ILSI: intralesional steroid injection

**Table S2.** Blood pressure change before and after procedure

| Case No | Baseline | Day 28  after the 1^st^- ILSI | Day 28  after the 2^nd^- ILSI | Day 28  after the 3^rd^-ILSI | Day 56  after the 3^rd^-ILSI | Day 84  after the 3^rd^-ILSI |
| --- | --- | --- | --- | --- | --- | --- |
| 2 | Normal | Normal | **Hypertension** | **High** | Normal | Normal |
| 3 | Normal | Normal | Normal | Normal | Normal | Normal |
| 4 | **High** | Normal  and fou and | **High** | **Hypertension** | **Hypertension** | **Hypertension** |
| 5 | Normal | **High** | **Hypertension** | **Hypertension** | **High** | **High** |
| 6 | Normal | Normal | Normal | Normal | Normal | Normal |
| 7 | Normal | **High** | Normal | **Hypertension** | Normal | **High** |
| 8 | **Hypertension** | **Hypertension** | Normal | **Hypertension** | **Hypertension** | **Hypertension** |
| 9 | Normal | Normal | Normal | Normal | Normal | Normal |

Normal blood pressure: systolic blood pressure 120-129 mmHg and/or diastolic blood pressure 80-84 mmHg,

High blood pressure: systolic blood pressure 130-139 mmHg and/or diastolic blood pressure 85-89 mmHg,

Hypertension: systolic blood pressure ≥ 140 mmHg and/or diastolic blood pressure ≥ 90 mmHg

**Table S3.** Fasting blood sugar before and after procedures

| Case No | Baseline | Day 28  after the 1^st^- ILSI | Day 28  after the 2^nd^- ILSI | Day 28  after the 3^rd^-ILSI | Day 56  after the 3^rd^-ILSI | Day 84  after 3^rd^-ILSI |
| --- | --- | --- | --- | --- | --- | --- |
| 2 | Normal | Normal | Normal | Normal | Normal | Normal |
| 3 | Normal | Normal | **IFG** | Normal | Normal | Normal |
| 4 | Normal | Normal  and fou and | Normal | Normal | Normal | Normal |
| 5 | Normal | Normal | **IFG** | Normal | Normal | Normal |
| 6 | Normal | **IFG** | **IFG** | **IFG** | **IFG** | Normal |
| 7 | Normal | Normal | Normal | Normal | Normal | Normal |
| 8 | Normal | Normal | Normal | **DM** | Normal | Normal |
| 9 | Normal | Normal | Normal | Normal | Normal | Normal |

Normal fasting blood sugar < 100; steroid induced impaired fasting glucose(IFG)=100-126;

steroid induced diabetes mellitus (DM) ≥ 126; ILSI: intralesional steroid injection

**Table S4.** Morning cortisol level and ACTH stimulation test

| **Case No** | **Morning cortisol** | **ACTH stimulation test baseline** | **ACTH stimulation test at 30 minutes** | **ACTH stimulation test at 60 minutes** |
| --- | --- | --- | --- | --- |
| **2*** | 5.6 | 5.6 | 15.92 | 15.02 |
| **3*** | 2.93 | - | - | - |
| 4 | 9 | 9.5 | 21.7 | 30.02 |
| 5 | 18.83 | - | - | - |
| **6*** | 7.9 | 7.9 | 9.59 | 15.14 |
| 7 | 20.7 | - | - | - |
| **8*** | 6.81 | 6.81 | 9.85 | 13.03 |
| 9 | 21.75 | - | - | - |

*****Presenting of hypothalamic-pituitary-adrenal axis suppression

**Figure S1.** Serum triamcinolone level 24-hours after intralesional steroid injection**.**

**
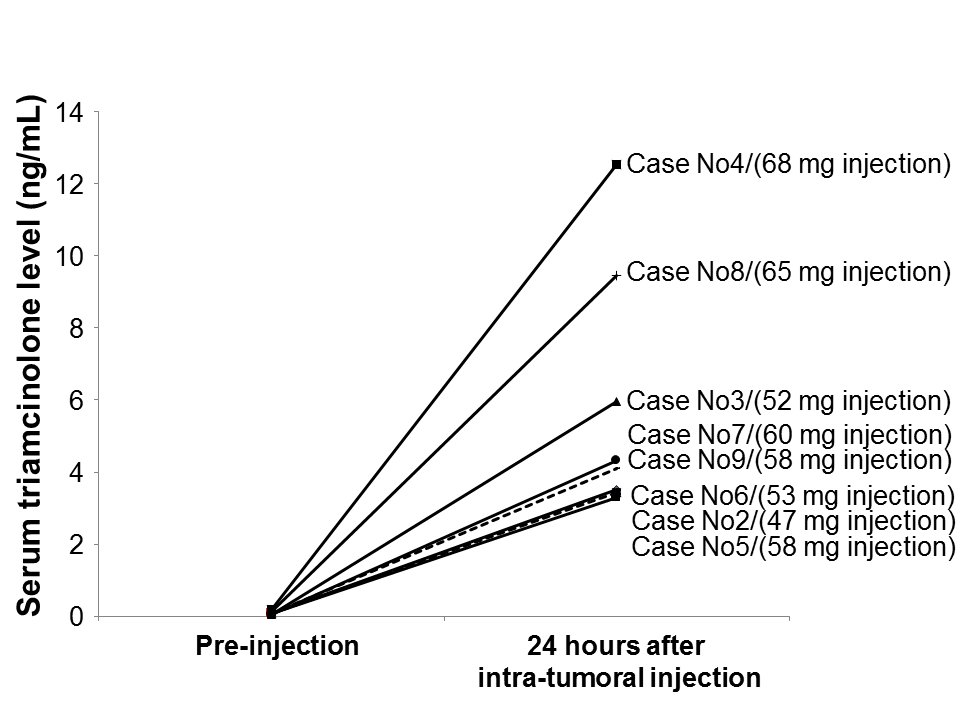
**
